# Supplementary material for: Red and Processed Meat Intake, Polygenic Risk Score, and Colorectal Cancer Risk
Source: Nutrients. 2022 Mar 3;14(5):1077. doi: 10.3390/nu14051077 (PMC8912739; doi:10.3390/nu14051077)
Supplement: Supplementary file 1 [file nutrients-14-01077-s001.zip › nutrients-1622555-supplementary.pdf]

## Supplementary Method

### Derivation of genetic risk equivalent (GRE) and confidence intervals (CIs) for GRE

GRE was developed based on a well-established concept of risk and rate advancement periods [1]. In brief, consider an analysis based on a multivariable logistic regression:

$$\ln(R) = a + b1 \cdot R + b2 \cdot P + \sum_{i=1}^{n} ci \cdot Fi$$

where ln(R) reflects the log odds of the disease risk, and a, $b_1$, $b_2$, and $c_i$ (i = 1, . . ., n) refer to the intercept and the model parameters for R (red and processed meat intake, categorized as 1 for the respective group who frequently consumed red and processed meat and 0 for the group consuming meat ≤1 time/week), P (PRS percentile according to the distribution among controls), and F (other covariates), respectively. GRE equals to the ratio of the estimated coefficients for red and processed meat intake categories and the PRS from the logistic regression models. Thus, the properties of GRE follow from the corresponding properties of $b_1$ and $b_2$, which include consistency, asymptotic unbiasedness, and normality. Using the delta method [2], the asymptotic variance of GRE can be derived as:

$$\mathrm{var}(GRE) = \frac{1}{{b_2}^2}\left[\mathrm{var}(b_1) - 2\cdot\left(\frac{b_1}{b_2}\right)\cdot \mathrm{cov}(b_1, b_2) + \left(\frac{b_1}{b_2}\right)^2 \cdot \mathrm{var}(b_2)\right]$$

As the GRE is asymptotically normal, its 95% CIs can be easily calculated using the square root of var(GRE) and sample size (n):

$$GRE \pm 1.96\sqrt{\frac{\mathrm{var}(GRE)}{n}}$$

**Table S1.** Reclassification of red and processed meat intake.

| Red Meat | Processed Meat | | | | | |
|---|---|---|---|---|---|---|
| | **Multiple times per day** | **Once per day** | **Multiple times per week** | **Once per week** | **Less than once per week** | **Never** |
| Multiple times per day | 4 | 4 | 4 | 4 | 4 | 4 |
| Once per day | 4 | 4 | 4 | 3 | 3 | 3 |
| Multiple times per week | 4 | 4 | 3 | 2 | 2 | 2 |
| Once per week | 4 | 3 | 2 | 1 | 1 | 1 |
| Less than once per week | 4 | 3 | 2 | 1 | 1 | 1 |
| Never | 4 | 3 | 2 | 1 | 1 | 1 |

NOTE: Red meat included fresh pork, beef, lamb, and sausages made from beef, pork; Processed meat included all luncheon meats (e.g., sliced sausage, salami) and ham. 1= ≤1 time/week (including never), 2= Multiple times/week, 3= 1 time/day, 4= >1 time/day.

**Table S2.** Information about genotyping and imputation.

| Genotyping platform | Cases (N) | Controls (N) | Recruitment Period | Imputation |
|---|---|---|---|---|
| Illumina HumanCytoSNP | 1705 | 1703 | 2003-2008 | Haplotype Reference Consortium (version r1.1.2016) |
| Illumina HumanOmniExpress | 665 | 494 | 2007-2010 | |
| Illumina HumanOmniExpress | 1186 | 626 | 2010-2015 | |
| Illumina Infinium OncoArray | 897 | 656 | 2003-2016 | |
| Illumina Global Screening Array | 656 | 655 | 2016-2017 | |

NOTE: We excluded triallelic SNPs, genotyped SNPs which had a low call rate (<98%), lack of Hardy-Weinberg equilibrium in control individuals ($p<1\times10^{-4}$), or low minor allele frequency (<0.1%), and those not assigned an rs number. More details can be found in the previous studies by Peters et al [3] and Schumacher et al [4].

**Table S3.** Overview on colorectal cancer related single-nucleotide polymorphisms that were identified in genome-wide association studies and considered in this analysis.

| SNP | Locus | Position | Risk allele | Beta |
|---|---|---|---|---|
| rs4360494 | 1p34.3 | 38455891 | G | 0.0379 |
| rs12144319 | 1p32.3 | 55246035 | C | 0.0661 |
| rs72647484 | 1p36.12 | 22587728 | T | 0.0504 |
| rs7542665 | 1p31.3 | 62673037 | C | 0.0334 |
| rs6678517 | 1q25.3 | 183002639 | A | 0.073 |
| rs17011141 | 1q41 | 222112634 | G | 0.0877 |
| rs448513 | 2q24.2 | 159964552 | C | 0.0054 |
| rs11884596 | 2q33.1 | 199612407 | C | 0.0342 |
| rs983402 | 2q33.1 | 199781586 | T | 0.0622 |
| rs7606562 | 2p16.3 | 48686695 | T | 0.0414 |
| rs11692435 | 2q11.2 | 98275354 | G | 0.0492 |
| rs3731861 | 2q35 | 219191256 | T | 0.0613 |
| rs10049390 | 3q22.2 | 133701119 | A | 0.0455 |
| rs13086367 | 3q13.2 | 112903888 | A | 0.0463 |
| rs72942485 | 3q13.2 | 112999560 | G | 0.0545 |
| rs9831861 | 3p21.1 | 53088285 | G | 0.0294 |
| rs35470271 | 3p22.1 | 40915239 | G | 0.0994 |
| rs12635946 | 3q13.2 | 112916918 | C | 0.0334 |
| rs113569514 | 3q22.2 | 133748789 | T | 0.0414 |
| rs9876206 | 3q26.2 | 169517436 | C | 0.0453 |
| rs6781752 | 3p14.1 | 66365163 | A | 0.0597 |
| rs11727676 | 4q31.21 | 145659064 | C | 0.0093 |
| rs1391441 | 4q24 | 106128760 | A | 0.0148 |
| rs13149359 | 4q22.2 | 94938618 | A | 0.052 |
| rs7708610 | 5p13.1 | 40102443 | A | 0.0384 |
| rs78368589 | 5p15.33 | 1240204 | T | 0.0786 |
| rs145364999 | 5q21.1 | 98206082 | T | 0.3496 |
| rs2735940 | 5p15.33 | 1296486 | G | 0.0865 |
| rs12514517 | 5p13.1 | 40280076 | A | 0.1013 |
| rs755229494 | 5q22.2 | 112097351 | G | 0.6286 |
| rs12659017 | 5q23.2 | 125988175 | G | 0.0374 |
| rs4976270 | 5q31.1 | 134467220 | C | 0.0693 |
| rs13204733 | 6p12.1 | 55566108 | G | 0.0643 |
| rs116685461 | 6p21.33 | 31315512 | G | 0.0655 |
| rs9271695 | 6p21.32 | 32593080 | G | 0.0889 |
| rs2516420 | 6p21.33 | 31449620 | C | 0.1091 |
| rs116353863 | 6p21.33 | 31010185 | C | 0.1202 |
| rs16878812 | 6p21.31 | 35569562 | A | 0.0778 |
| rs9470361 | 6p21.2 | 36623379 | A | 0.054 |
| rs62404966 | 6p12.1 | 55712124 | C | 0.0724 |
| rs3131043 | 6p21.33 | 30758466 | G | 0.0294 |
| rs2070699 | 6p24.1 | 12292772 | T | 0.0294 |
| rs1476570 | 6p22.1 | 29809860 | A | 0.0492 |
| rs3830041 | 6p21.32 | 32191339 | T | 0.0645 |
| rs6928864 [1] | 6q21 | 105966894 | C | 0.0531 |
| rs62396735 | 6p21.1 | 41702582 | C | 0.033 |
| rs12672022 | 7p13 | 45136423 | T | 0.0067 |
| rs80077929 | 7p12.3 | 46094089 | T | 0.0093 |

| | | | | |
|---|---|---|---|---|
| rs10951878 | 7p12.3 | 46926695 | C | 0.0531 |
| rs3801081 | 7p12.3 | 47511161 | G | 0.0253 |
| rs7013278 | 8q24.21 | 128414892 | T | 0.0091 |
| rs4313119 | 8q24.21 | 128571855 | G | 0.0518 |
| rs16892766 | 8q23.3 | 117630683 | C | 0.2099 |
| rs6469654 | 8q23.3 | 117632965 | G | 0.0677 |
| rs117079142 | 8q24.11 | 117790914 | A | 0.1139 |
| rs6983267 | 8q24.21 | 128413305 | G | 0.1052 |
| rs34405347 | 9q22.33 | 101679752 | T | 0.0089 |
| rs1537372 | 9p21.3 | 22103183 | G | 0.012 |
| rs10980628 | 9q31.3 | 113671403 | C | 0.0511 |
| rs12217641 | 10p14 | 8663875 | C | 0.0069 |
| rs10786560 | 10q24.2 | 101315166 | G | 0.0082 |
| rs1250567 | 10q22.3 | 81046265 | C | 0.047 |
| rs11255841 | 10p14 | 8739580 | T | 0.1064 |
| rs10821907 | 10q11.23 | 52648454 | C | 0.073 |
| rs704017 | 10q22.3 | 80819132 | G | 0.0765 |
| rs11190164 | 10q24.2 | 101351704 | G | 0.0889 |
| rs12246635 | 10q25.2 | 114288619 | C | 0.0975 |
| rs11196170 | 10q25.2 | 114722621 | A | 0.0527 |
| rs7946853 | 11q13.4 | 74409077 | C | 0.0119 |
| rs55864876 | 11q22.1 | 100717136 | G | 0.015 |
| rs2186607 | 11q22.1 | 101656397 | T | 0.0483 |
| rs61389091 | 11q13.4 | 74427921 | C | 0.1934 |
| rs4450168 | 11p15.4 | 10286755 | C | 0.0413 |
| rs174533 | 11q12.2 | 61549025 | G | 0.0636 |
| rs7121958 | 11q13.4 | 74280012 | G | 0.078 |
| rs3087967 | 11q23.1 | 111156836 | T | 0.1122 |
| rs4759277 | 12q13.3 | 57533690 | A | 0.0285 |
| rs1427760 | 12q24.21 | 115100714 | C | 0.0424 |
| rs3217874 | 12p13.32 | 4400808 | T | 0.0453 |
| rs10849433 | 12p13.31 | 6406904 | C | 0.0468 |
| rs11610543 | 12q12 | 43134191 | G | 0.0474 |
| rs35808169 | 12p13.32 | 4368607 | C | 0.089 |
| rs3217810 | 12p13.32 | 4388271 | T | 0.1181 |
| rs2250430 | 12p13.31 | 6421174 | T | 0.0597 |
| rs77969132 | 12p11.21 | 31594813 | T | 0.1583 |
| rs12372718 | 12q13.12 | 51171090 | G | 0.0896 |
| rs597808 | 12q24.12 | 111973358 | G | 0.0737 |
| rs7300312 | 12q24.21 | 115890922 | C | 0.066 |
| rs2710310 | 12p13.2 | 12035649 | C | 0.0145 |
| rs78341008 | 13q22.1 | 73791554 | C | 0.0109 |
| rs8000189 | 13q34 | 111075881 | T | 0.0473 |
| rs45597035 | 13q22.1 | 73649152 | A | 0.0495 |
| rs1924816 | 13q22.1 | 73997961 | A | 0.0506 |
| rs7333607 | 13q13.3 | 37462010 | G | 0.0758 |
| rs1330889 | 13q22.3 | 78609615 | C | 0.0453 |
| rs377429877 | 13q13.2 | 34092164 | C | 0.0468 |
| rs1951864 | 14q22.2 | 54369299 | A | 0.0059 |
| rs17094983 | 14q23.1 | 59189361 | G | 0.0062 |
| rs8020436 | 14q23.1 | 59208437 | A | 0.0294 |

| | | | | |
|---|---|---|---|---|
| rs35107139 | 14q22.2 | 54419106 | C | 0.0912 |
| rs4901473 | 14q22.2 | 54445157 | G | 0.0465 |
| rs745213 | 15q23 | 68060389 | G | 0.0072 |
| rs12594720 | 15q22.31 | 67007018 | C | 0.0246 |
| rs56324967 | 15q22.33 | 67402824 | C | 0.0689 |
| rs17816465 | 15q13.3 | 33156386 | A | 0.069 |
| rs12708491 | 15q13.3 | 32992836 | G | 0.0464 |
| rs2293581 | 15q13.3 | 33010736 | A | 0.1248 |
| rs7495132 | 15q26.1 | 91172901 | T | 0.0453 |
| rs9930005 | 16q23.2 | 80043258 | C | 0.0061 |
| rs12447408 | 16q24.1 | 86252544 | A | 0.0079 |
| rs9924886 | 16q22.1 | 68743939 | A | 0.055 |
| rs12149163 | 16q24.1 | 86339315 | T | 0.0487 |
| rs62042090 | 16q24.1 | 86703949 | T | 0.0481 |
| rs983318 | 17q24.3 | 70413253 | A | 0.0397 |
| rs73975586 | 17p13.3 | 814243 | A | 0.0497 |
| rs1078643 | 17p12 | 10707241 | A | 0.0747 |
| rs75954926 | 17q25.3 | 81061048 | G | 0.0882 |
| rs373585858 | 17q25.3 | 80394556 | A | 0.1103 |
| rs4968127 | 17p13.3 | 809643 | G | 0.0514 |
| rs11874392 | 18q21.1 | 46453156 | A | 0.1606 |
| rs73068325 | 19q13.43 | 59079096 | T | 0.0066 |
| rs34797592 | 19p13.11 | 16417198 | T | 0.0824 |
| rs28840750 | 19q13.11 | 33519927 | T | 0.1939 |
| rs1963413 | 19q13.2 | 41871573 | A | 0.0441 |
| rs12979278 | 19q13.33 | 49218602 | T | 0.0293 |
| rs2738783 | 20q13.33 | 62308612 | T | 0.006 |
| rs6067417 | 20q13.13 | 48983697 | C | 0.0331 |
| rs6031311 | 20q13.12 | 42666475 | T | 0.0362 |
| rs6091189 | 20q13.13 | 49256285 | T | 0.0549 |
| rs994308 | 20p12.3 | 6603622 | C | 0.0626 |
| rs28488 | 20p12.3 | 6762221 | T | 0.0714 |
| rs556532366 | 20p12.3 | 8568071 | T | 0.0715 |
| rs189583 | 20p12.3 | 6376457 | G | 0.0795 |
| rs4813802 | 20p12.3 | 6699595 | G | 0.0819 |
| rs11087784 | 20p12.3 | 7740976 | G | 0.0874 |
| rs6066825 | 20q13.13 | 47340117 | A | 0.0719 |
| rs6063514 | 20q13.13 | 49055318 | C | 0.0547 |
| rs13831 | 20q13.32 | 57475191 | G | 0.0334 |
| rs1741640 | 20q13.33 | 60932414 | C | 0.1146 |
| rs6058093 | 20q11.22 | 33213196 | C | 0.045 |

[1] For building the PRS, the missing reference SNP was replaced by rs6904092 (linkage disequilibrium, D′=1 and $r^2$=1). Abbreviations: A, adenine; C, cytosine; G, guanine; OR, odds ratio; T, thymine; SNP, single-nucleotide polymorphism.

**Table S4.** Association of red and processed meat intake and CRC risk in different subgroups.

| Subgroups | Red and processed meat intake | Cases, N (%) | Controls, N(%) | OR (95% CI) [1] | GRE (95% CI) | p-interaction [2] |
|---|---|---|---|---|---|---|
| Age | | | | | | 0.45 |
| ≤55 years | ≤1 time/week | 56 (8.8) | 53 (11.1) | Ref. | Ref. | |
| | Multiple times/week | 356 (55.7) | 298 (62.2) | 0.95 (0.60, 1.50) | -4.2 (-41.9, 33.5) | |
| | 1 time/day | 183 (28.6) | 108 (22.5) | 1.28 (0.77, 2.13) | 20.2 (-21.6, 62.0) | |
| | >1 time/day | 44 (6.9) | 20 (4.2) | 1.43 (0.69, 3.00) | 29.3 (-31.0, 89.6) | |
| | | | | | | |
| >55 years | ≤1 time/week | 331 (7.8) | 416 (11.8) | Ref. | Ref. | |
| | Multiple times/week | 2569 (60.4) | 2146 (60.7) | 1.24 (1.04, 1.49) | 16.4 (2.7, 30.2) | |
| | 1 time/day | 1175 (27.6) | 871 (24.6) | 1.45 (1.19, 1.76) | 28.4 (13.0, 43.7) | |
| | >1 time/day | 178 (4.2) | 104 (2.9) | 1.75 (1.28, 2.41) | 42.7 (17.9, 67.6) | |
| Sex | | | | | | 0.47 |
| Female | ≤1 time/week | 264 (13.7) | 287 (18.6) | Ref. | Ref. | |
| | Multiple times/week | 1230 (63.9) | 976 (63.2) | 1.19 (0.96, 1.46) | 13.3 (-2.9, 29.4) | |
| | 1 time/day | 391 (20.3) | 255 (16.5) | 1.51 (1.17, 1.94) | 31.5 (11.5, 51.8) | |
| | >1 time/day | 39 (2.0) | 26 (1.7) | 1.31 (0.73, 2.37) | 20.6 (-24.2, 65.4) | |
| | | | | | | |
| Male | ≤1 time/week | 123 (4.1) | 182 (7.4) | Ref. | Ref. | |
| | Multiple times/week | 1695 (57.1) | 1468 (59.4) | 1.24 (0.95, 1.63) | 16.4 (-4.4, 37.3) | |
| | 1 time/day | 967 (32.6) | 724 (29.3) | 1.43 (1.08, 1.89) | 27.3 (5.4, 49.2) | |
| | >1 time/day | 183 (6.2) | 98 (4.0) | 1.93 (1.32, 2.81) | 50.2 (20.4, 80.0) | |
| Family history of CRC | | | | | | 0.23 |
| No | ≤1 time/week | 321 (7.7) | 400 (11.2) | Ref. | Ref. | |
| | Multiple times/week | 2500 (59.9) | 2193 (61.3) | 1.17 (0.98, 1.40) | 12.8 (-1.9, 27.6) | |
| | 1 time/day | 1160 (27.8) | 877 (24.5) | 1.41 (1.16, 1.71) | 28.1 (11.6, 44.6) | |
| | >1 time/day | 195 (4.7) | 107 (3.0) | 1.89 (1.39, 2.59) | 52.1 (25.6, 78.5) | |
| | | | | | | |
| Yes | ≤1 time/week | 66 (9.2) | 69 (15.7) | Ref. | Ref. | |
| | Multiple times/week | 425 (59.4) | 251 (57.2) | 1.30 (0.83, 2.02) | 15.1 (-10.7, 40.8) | |
| | 1 time/day | 198 (27.7) | 102 (23.2) | 1.37 (0.83, 2.24) | 18.1 (-10.6, 46.8) | |
| | >1 time/day | 27 (3.8) | 17 (3.9) | 0.85 (0.38, 1.91) | -9.3 (-55.5, 36.9) | |
| History of colonoscopy | | | | | | 0.40 |
| No | ≤1 time/week | 271 (7.5) | 181 (11.3) | Ref. | Ref. | |
| | Multiple times/week | 2159 (60.0) | 981 (61.4) | 1.20 (0.96, 1.49) | 13.0 (-2.8, 28.9) | |
| | 1 time/day | 992 (27.6) | 385 (24.1) | 1.38 (1.08, 1.76) | 23.0 (5.4, 40.7) | |
| | >1 time/day | 177 (4.9) | 51 (3.2) | 2.04 (1.39, 3.02) | 51.0 (22.4, 79.7) | |
| | | | | | | |
| Yes | ≤1 time/week | 116 (9.0) | 288 (11.9) | Ref. | Ref. | |
| | Multiple times/week | 766 (59.2) | 1463 (60.5) | 1.19 (0.93, 1.53) | 15.3 (-6.8, 37.5) | |
| | 1 time/day | 366 (28.3) | 594 (24.6) | 1.45 (1.11, 1.90) | 32.8 (7.9, 57.7) | |
| | >1 time/day | 45 (3.5) | 73 (3.0) | 1.43 (0.90, 2.26) | 31.6 (-9.6, 72.7) | |

[1] Adjusted for age, sex, school education, body mass index, smoking, alcohol consumption, history of colonoscopy, history of diabetes, family history of CRC, use of statins, use of non-steroidal anti-inflammatory drugs, fish, whole grains, vegetables, fruits, dairy foods, and polygenic risk score (per 10 percentiles, continuous variable) but excluding stratification factors (age, sex, history of colonoscopy, or family history of CRC). [2] Interactions were tested by including a cross-product term of red and processed meat intake and stratification factors in models. Abbreviation: CI, confidence interval; CRC, colorectal cancer; GRE, genetic risk equivalent; OR, odds ratio; Ref., reference.

## Supplementary References


1. Brenner, H.; Gefeller, O.; Greenland, S. Risk and rate advancement periods as measures of exposure impact on the occurrence of chronic diseases. *Epidemiology* **1993**, *4*, 229–236, doi:10.1097/00001648-199305000-00006.
2. Bishop YMM, Fienberg SE, H.P. Discrete Multivariate Analysis: Theory and Practice.; MIT press, 1975.
3. Peters, U.; Jiao, S.; Schumacher, F.R.; Hutter, C.M.; Aragaki, A.K.; Baron, J.A.; Berndt, S.I.; Bézieau, S.; Brenner, H.; Butterbach, K.; et al. Identification of Genetic Susceptibility Loci for Colorectal Tumors in a Genome-Wide Meta-analysis. *Gastroenterology* **2013**, *144*, 799-807.e24, doi:10.1053/j.gastro.2012.12.020.
4. Schumacher, F.R.; Schmit, S.L.; Jiao, S.; Edlund, C.K.; Wang, H.; Zhang, B.; Hsu, L.; Huang, S.-C.; Fischer, C.P.; Harju, J.F.; et al. Genome-wide association study of colorectal cancer identifies six new susceptibility loci. *Nat. Commun.* **2015**, *6*, 7138, doi:10.1038/ncomms8138.
